# Systemic oxidative stress associates with disease severity and outcome in patients with new-onset or worsening heart failure

**DOI:** 10.1007/s00392-023-02171-x

**Published:** 2023-03-30

**Authors:** Marie-Sophie L. Y. de Koning, Johanna E. Emmens, Esteban Romero-Hernández, Arno R. Bourgonje, Solmaz Assa, Sylwia M. Figarska, John G. F. Cleland, Nilesh J. Samani, Leong L. Ng, Chim C. Lang, Marco Metra, Gerasimos S. Filippatos, Dirk J. van Veldhuisen, Stefan D. Anker, Kenneth Dickstein, Adriaan A. Voors, Erik Lipsic, Harry van Goor, Pim van der Harst

**Affiliations:** 1 Department of Cardiology, University of Groningen, University Medical Center Groningen, Hanzeplein 1, PO Box 30.001, 9700 RB Groningen, The Netherlands; 2 Faculty of Medicine, Institute of Biomedical Science, University of Chile, Santiago, Chile; 3 Department of Gastroenterology and Hepatology, University of Groningen, University Medical Center Groningen, Groningen, The Netherlands; 4 National Heart and Lung Institute, Royal Brompton and Harefield Hospitals, Imperial College, London, UK; 5 Robertson Centre for Biostatistics and Clinical Trials, University of Glasgow, Glasgow, UK; 6 Department of Cardiovascular Sciences, University of Leicester, NIHR Leicester Biomedical Research Centre, Glenfield Hospital, Leicester, UK; 7 Division of Molecular and Clinical Medicine, School of Medicine, University of Dundee, Dundee, UK; 8 Department of Medical and Surgical Specialties, Radiological Sciences and Public Health, Institute of Cardiology, University of Brescia, Brescia, Italy; 9 School of Medicine, National and Kapodistrian University of Athens, Athens, Greece; 10 Department of Cardiology (CVK), Center for Regenerative Therapies (BCRT), German Centre for Cardiovascular Research (DZHK) Partner Site Berlin, Berlin Institute of Health, Charité Universitätsmedizin Berlin, Berlin, Germany; 11 University of Bergen, Stavanger University Hospital, Bergen, Norway; 12 Department of Pathology and Medical Biology, University of Groningen, University Medical Center Groningen, Groningen, The Netherlands; 13 Division Heart and Lungs, Department of Cardiology, University Medical Center Utrecht, Utrecht, The Netherlands

**Keywords:** Heart failure, Thiols, Oxidative stress, Redox status, Sulfhydryl groups

## Abstract

**Background:**

Oxidative stress may be a key pathophysiological mediator in the development and progression of heart failure (HF). The role of serum-free thiol concentrations, as a marker of systemic oxidative stress, in HF remains largely unknown.

**Objective:**

The purpose of this study was to investigate associations between serum-free thiol concentrations and disease severity and clinical outcome in patients with new-onset or worsening HF.

**Methods:**

Serum-free thiol concentrations were determined by colorimetric detection in 3802 patients from the BIOlogy Study to TAilored Treatment in Chronic Heart Failure (BIOSTAT-CHF). Associations between free thiol concentrations and clinical characteristics and outcomes, including all-cause mortality, cardiovascular mortality, and a composite of HF hospitalization and all-cause mortality during a 2-years follow-up, were reported.

**Results:**

Lower serum-free thiol concentrations were associated with more advanced HF, as indicated by worse NYHA class, higher plasma NT-proBNP (*P* < 0.001 for both) and with higher rates of all-cause mortality (hazard ratio (HR) per standard deviation (SD) decrease in free thiols: 1.253, 95% confidence interval (CI): 1.171–1.341, *P* < 0.001), cardiovascular mortality (HR per SD: 1.182, 95% CI: 1.086–1.288, *P* < 0.001), and the composite outcome (HR per SD: 1.058, 95% CI: 1.001–1.118, *P* = 0.046).

**Conclusions:**

In patients with new-onset or worsening HF, a lower serum-free thiol concentration, indicative of higher oxidative stress, is associated with increased HF severity and poorer prognosis. Our results do not prove causality, but our findings may be used as rationale for future (mechanistic) studies on serum-free thiol modulation in heart failure.

**Graphical abstract:**

Associations of serum-free thiol concentrations with heart failure severity and outcomes
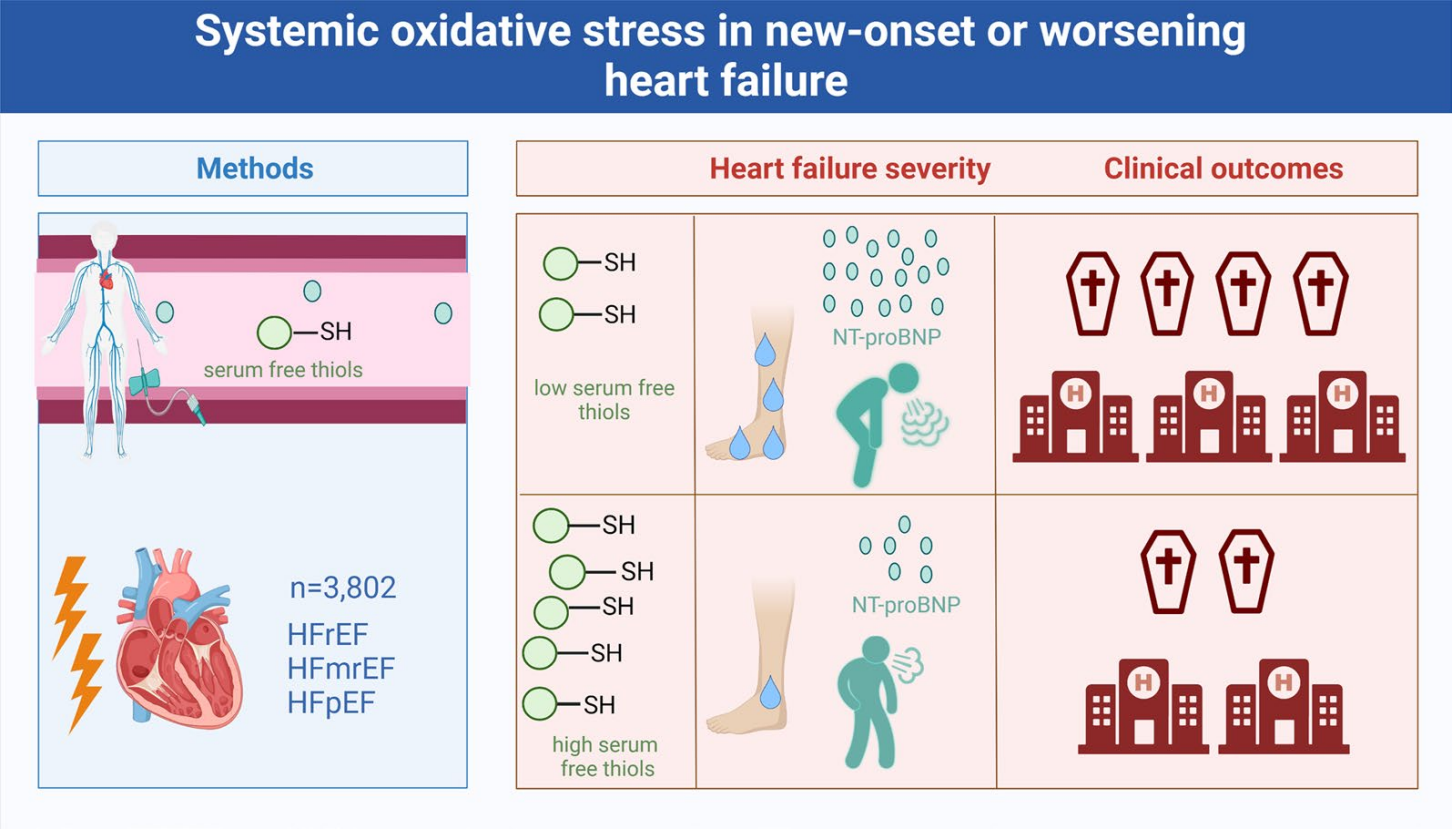

**Supplementary Information:**

The online version contains supplementary material available at 10.1007/s00392-023-02171-x.

## Introduction

Oxidative stress has been identified as an important pathophysiological mediator in the development and progression of heart failure (HF) [1, 2]. Oxidative stress reflects an imbalance between the production of reactive oxygen species (ROS) and the antioxidant capacity [3]. Although ROS provide important beneficial physiological functions at lower concentrations, excess of ROS can cause DNA damage and harmful modifications of proteins. This can result in cellular dysfunction, including changes in cardiomyocyte excitation–contraction coupling, calcium handling and energy metabolism [1]. Excess of ROS can also exert pro-fibrotic effects, leading to extracellular matrix remodeling and eventually worsening of diastolic and systolic function [4–6].

Free thiols, organosulfur compounds with an –SH group, act as one of the most potent and versatile endogenous defense mechanisms against oxidative stress. Extracellular, i.e. circulating, thiols are the sum of and high- and low-molecular-weight thiols and are referred to as total *free thiols.* Free thiols mainly comprise of high molecular weight proteins with an –SH group attached, of which albumin is the most relevant example, whereas circulating low-molecular weight thiols such as cysteine or glutathione only account for < 3–5% [7]. By forming stable disulfide bonds through ROS scavenging, free thiols prevent ROS from inflicting lipid and protein oxidation and subsequent myocardial structural damage [7]. Depletion of the antioxidant-free thiol pool reflects greater oxidative stress, and has been linked to the severity of oxidative stress-associated diseases [8, 9]. Increasing free thiol levels (e.g., by *N*-acetylcysteine) improved cardiac function in animal models of HF and cardiac injury [10–13]. In addition, clinical trials in small numbers of patients with myocardial infarction or HF suggested that *N*-acetylcysteine may reduce oxidative stress [14–16]. Hence, targeting thiol levels may hold promise as a potential therapeutic strategy in patients with HF. Accordingly, we investigated the associations between serum-free thiol concentrations and clinical characteristics and outcomes in a large cohort of patients with new-onset or worsening HF including a broad range of left ventricular phenotypes.

## Methods

### Study population

We measured the concentration of free thiols in archived serum samples of the multinational, prospective, observational BIOSTAT-CHF study. Study design and data collection have been described in full elsewhere [17]. In brief, in the BIOSTAT-CHF index cohort, 2516 patients with new-onset or worsening signs and/or symptoms of HF from 11 European countries were included between 2010 and 2012. Participants were documented with a left ventricular ejection fraction (LVEF) of ≤ 40% or plasma N-terminal pro-B-type natriuretic peptide (NT-proBNP) of > 2000 ng/L. In addition, participants were considered to be on suboptimal evidence-based treatment for HF before enrollment [18]. A comparable validation cohort of BIOSTAT-CHF included another 1738 patients from six centers in Scotland between 2010 and 2014, who had to have a previously documented admission for HF. No additional NT-proBNP criteria were used for patients with a LVEF > 40% in the validation cohort, which resulted in a higher percentage of patients with a LVEF > 45%: 34% in the validation cohort versus 7% in the index cohort. The BIOSTAT-CHF study was conducted according to the Declaration of Helsinki, approved by the ethics committee of each center and all participants provided written informed consent prior to any study-related procedures.

### Detection of serum-free thiols

Blood samples were drawn upon enrollment in the BIOSTAT-CHF cohort. Serum samples were stored at − 80 °C until free thiol measurement. The free thiol concentration was detected as previously described, with minor modifications [19, 20]. In short, after thawing, 75 μl serum samples were diluted 1:4 with a 0.1 M Tris buffer (pH 8.2) and then transferred to a microplate. The background absorption was measured, using a Sunrise microplate reader (Tecan Trading AG, Männedorf, Switzerland) at 412 nm, with a reference filter at 630 nm. Subsequently, 10 μl 3.8 mM 5,5′-Dithio-bis(2-nitrobenzoic acid) (DTNB, CAS-number 69–78–3, Sigma Aldrich Corporation, Saint Louis, MO, USA) in a 0.1 M phosphate buffer (pH 7.0) was added to the samples. Following 20 min of incubation at room temperature, absorption was measured again and subtracted from background absorption. The concentration of free thiols in the samples was determined by parallel measurement of an l-cysteine (CAS-number 52–90–4, Fluka Biochemika, Buchs, Switzerland) calibration standard in the concentration range of 15.6–1000 μM in 0.1 M Tris and 10 mM EDTA (pH 8.2). All measurements were performed *in duplo*, where the mean of the free thiol value of both measurements was used for analyses. Measurements with a coefficient of variation > 20% were excluded from further analysis.

### Clinical outcome parameters

The primary clinical outcome parameter of this study was a composite endpoint of all-cause mortality and HF-related hospitalizations at 2 years. Our secondary outcome was all-cause mortality at 2 years. As sensitivity analysis, associations with cardiovascular mortality were studied. The cause of death and hospitalization were determined by the individual site investigators. Clinical events were collected during the 9-months follow-up study visit (index cohort), standard clinical follow-up and by telephonic contacts every 6 months for at least 2 years or until the end of follow-up (2015).

### Statistical analysis

For this study, both BIOSTAT-CHF cohorts were analyzed together. Normally distributed variables were displayed as mean with standard deviation (SD), non-normally distributed variables as median with interquartile range [IQR], and categorical variables as numbers with percentages (%). Distribution of continuous data was visually inspected using normal probability (Q–Q) plots. Baseline characteristics were presented according to tertiles of serum-free thiol levels. Between-group differences were compared using one-way analysis of variance (ANOVA), the Kruskal–Wallis test or the chi-square test, as appropriate. Clinical characteristics with a *P*-value < 0.1 were selected from the baseline table to investigate their associations with the serum-free thiol concentration using univariable, age- and sex-adjusted and multivariable linear regression analyses. All variables with *P* < 0.1 in age- and sex-adjusted analyses were included in multivariable analysis and subjected to backward elimination. Variables with *P* < 0.05 were retained in the final multivariable regression model. Prior to linear regression, normal distribution of residuals was checked, as well as presence of outliers. All variables were standardized and non-normally distributed variables were log-transformed before entry into regression analysis. To assess associations with clinical outcomes at 2-years follow-up, follow-up time and clinical events were truncated at 730.5 days. Kaplan–Meier survival curves were drawn for tertiles of serum-free thiols. The log-rank test was used to test for differences in outcomes between the tertiles. Subsequently, Cox proportional hazards regression analyses were performed to investigate associations between the free thiol concentration and disease outcome. The proportionality of hazards assumption was checked for all models to confirm absence of violation. Cox regression analyses were adjusted in a stepwise manner by first adjusting for age and sex and subsequently for sex and the previously published BIOSTAT-CHF risk models [21]. Variables in BIOSTAT-CHF risk model to predict the composite endpoint included age, HF hospitalization in the year before inclusion, edema, NT-proBNP, systolic blood pressure, hemoglobin, high-density lipoprotein levels, serum sodium concentration, and the failure to prescribe a beta-blocker. When investigating the associations between free thiols and cardiovascular mortality, non-cardiovascular mortality was used as competing risk. For the primary and secondary endpoint, pre-specified subgroup analyses were performed, testing for interactions for age (≤ 70 vs > 70), sex, HF groups (HF with reduced ejection fraction (HFrEF) vs HF with mildly reduced ejection fraction (HFmrEF) vs HFpEF), ischemic etiology, NYHA class (I–II vs III–IV) and history of chronic kidney disease (CKD). In this study, a *P*-value of < 0.05 was considered statistically significant. All analyses were conducted with R version 3.5.2 (R Foundation for Statistical Computing, Vienna, Austria).

## Results

### Patient characteristics

Serum-free thiol levels were measured in 3802 participants of the BIOSTAT-CHF cohort. Mean free thiol concentration was 336 (SD 92) µmol/L. Baseline characteristics of the study population are presented according to tertiles of free thiol levels in Table 1. Patients within the lowest tertile were older (75 vs 69 years old, *P* < 0.001), more often female (36% vs 23%, *P* < 0.001), had less frequent an ischemic etiology of HF (56 vs 64%, *P* < 0.001) and shorter duration of HF diagnosis (median 10 vs 15 months, *P* = 0.003), compared with patients within the highest tertile of free thiol levels. Patients within the lowest tertile experienced more signs and symptoms of HF, had more advanced NYHA classes and higher NT-proBNP levels (*P* < 0.001 for all). Baseline characteristics are also presented for both cohorts separately (Supplementary Tables 1 and 2). The distribution of characteristics across the free thiols tertiles within the individual cohorts was quite comparable to the distribution in the combined study population.

**Table 1:** Baseline characteristics of the study population according to tertiles of serum-free thiol concentrations

	1st tertile *n* = 1268	2nd tertile *n* = 1267	3rd tertile *n* = 1267	*P*-value
Serum-free thiols (μmol/L)	246 [204;273] Full range (38–298)	339 [320;359] Full range (298–378)	423 [399;459] Full range (379–799)	
*Demographics*				
Age (years)	75.2 [66.9;81.5]	73.4 [64.1;80.4]	68.7 [59.7;76.5]	** < 0.001**
Female sex	450 (36%)	403 (32%)	293 (23%)	** < 0.001**
BMI (kg/m^2^)	27.1 [23.8;31.1]	27.3 [24.1;31.6]	27.8 [24.6;31.9]	0.007
HF type				0.52
HFrEF	753 (66%)	746 (65%)	747 (66%)	
HFmrEF	197 (17%)	207 (18%)	223 (20%)	
HFpEF	188 (17%)	187 (16%)	167 (15%)	
Months since HF diagnosis	10 [0;47]	19 [1;59]	15 [2;58]	**0.003**
Ischemic etiology	632 (56%)	701 (65%)	673 (64%)	** < 0.001**
Inpatient at enrollment	1004 (79%)	789 (62%)	628 (50%)	** < 0.001**
NYHA class				** < 0.001**
I/II	372 (30%)	489 (39%)	629 (50%)	
III/IV	868 (70%)	756 (61%)	623 (50%)	
Systolic BP (mmHg)	120 [108;135]	124 [110;140]	123 [110;140]	** < 0.001**
Diastolic BP (mmHg)	70 [60;80]	70 [63;80]	72 [65;80]	** < 0.001**
Heart rate (bpm)	75 [65;88]	74 [64;87]	74 [65;85]	**0.033**
LVEF (%)	35 [25;43]	35 [25;43]	35 [25;43]	0.97
*Signs and symptoms*				
Peripheral edema	790 (71%)	652 (60%)	528 (50%)	** < 0.001**
Elevated JVP	372 (38%)	302 (30%)	233 (24%)	** < 0.001**
Hepatomegaly	165 (14%)	106 (9%)	92 (8%)	** < 0.001**
Pulmonary congestion	746 (61%)	613 (50%)	466 (38%)	** < 0.001**
*Medical history*				
Anemia	560 (45%)	465 (38%)	342 (28%)	** < 0.001**
Atrial fibrillation	618 (49%)	576 (46%)	508 (40%)	** < 0.001**
Diabetes Mellitus	404 (32%)	425 (34%)	404 (32%)	0.60
COPD	242 (19%)	233 (19%)	193 (15%)	**0.025**
CKD	561 (44%)	470 (37%)	319 (26%)	** < 0.001**
Hypertension	798 (63%)	747 (59%)	751 (59%)	0.08
PAVD	181 (14%)	194 (16%)	222 (18%)	0.07
Stroke	179 (14%)	188 (15%)	133 (11%)	**0.002**
PCI	217 (17%)	250 (20%)	287 (23%)	**0.002**
CABG	221 (17%)	214 (17%)	220 (17%)	0.93
*Medicines*				
Loop diuretics	1259 (99%)	1256 (99%)	1256 (99%)	0.88
Loop diuretic dose (mg furosemide equivalent)	50 [40;120]	40 [40;80]	40 [40;80]	** < 0.001**
ACEi/ARB	860 (68%)	880 (70%)	978 (77%)	** < 0.001**
Beta-blocker	985 (78%)	989 (78%)	1017 (80%)	0.23
MRA	574 (45%)	549 (43%)	542 (43%)	0.42
*Laboratory data*				
Hemoglobin (g/dL)	12.8 [11.4;14.1]	13.2 [11.8;14.5]	13.7 [12.5;14.9]	** < 0.001**
Leucocytes (10^9^/L)	7.8 [6.3:9.7]	7.7 [6.3;9.4]	7.5 [6.2;9.2]	**0.015**
Sodium (mmol/L)	139 [136;141]	139 [137;141]	140 [138;141]	** < 0.001**
Potassium (mmol/L)	4.2 [3.9;4.6]	4.3 [4.0;4.6]	4.3 [4.0;4.6]	**0.007**
Urea (mmol/L)	11.8 [8.1;19.3]	9.5 [7.0;14.1]	8.4 [6.2;12.1]	** < 0.001**
Serum creatinine (μmol/L)	111 [88;146]	101 [82;128]	93 [78;114]	** < 0.001**
eGFR (mL/min/1.73 m^2^)	52 [37;60]	59 [44;62]	60 [54;69]	** < 0.001**
NT-proBNP (ng/L)	3466 [1398;7861]	2212 [944;4810]	1208 [479;2936]	** < 0.001**
NT-proBNP in sinus rhythm	2823 [1039; 7584]	1746 [601;4058]	921 [339;2479]	** < 0.001**
NT-proBNP in atrial fibrillation/flutter	3953 [2022; 8127]	2763 [1492;5043]	1789 [933;3578]	** < 0.001**
Albumin (g/L)	31 [26;36]	36 [31;40]	38 [33;42]	** < 0.001**
LDL-cholesterol (mmol/L)	2.1 [1.6;2.7]	2.1 [1.6;2.9]	2.3 [1.7;3.0]	**0.003**
HDL-cholesterol (mmol/L)	1.06 [0.83;1.34]	1.09 [0.90;1.35]	1.08 [0.86;1.33]	0.19
Glucose (mmol/L)	6.4 [5.4;8.1]	6.3 [5.4;8.3]	6.1 [5.2;8.1]	**0.015**

Data shown as median [IQR] or *n* (%). Significant *P*-values are bold-printed

*ACEi* angiotensin-converting enzyme inhibitor, *ARB* angiotensin receptor blocker, *BMI* body mass index, *BP* blood pressure, *CABG* coronary artery bypass graft, *COPD* chronic obstructive pulmonary disease, *CKD* chronic kidney disease, *eGFR* estimated glomerular filtration rate, *HDL* high-density lipoprotein, *HF* heart failure, *HFmrEF* heart failure with mildly reduced ejection fraction, *HFpEF* heart failure with preserved ejection fraction, *HFrEF* heart failure with reduced ejection fraction, *IQR* interquartile range, *JVP* jugular venous pressure, *LDL* low-density lipoprotein, *LVEF* left ventricular ejection fraction, *MRA* mineralocorticoid receptor antagonist, *NT-proBNP* N-terminal pro-B-type natriuretic peptide, *NYHA* New York Heart Association, *PAVD* peripheral arterial vascular disease, *PCI* percutaneous coronary intervention

Associations between free thiol concentration and age, sex, signs and symptoms of HF and NT-proBNP were also observed in regression analyses (Table 2). Moreover, lower free thiol levels were associated with lower systolic and diastolic blood pressure, enrollment in the BIOSTAT-CHF cohort as an inpatient (instead of at the outpatient clinic), and the absence of treatment with an angiotensin-converting enzyme inhibitor (ACEi) or angiotensin receptor blocker (ARB). Furthermore, (laboratory parameters of) other diseases, including anemia and chronic kidney disease were associated with lower levels of serum-free thiols. Lower albumin, higher age, and higher urea and NT-proBNP were the strongest determinants of lower free thiol concentration (*P* < 0.001 for all; Table 2).

**Table 2:** Clinical characteristics and laboratory values associated with serum-free thiols

	*n* =	Age- and sex-adjusted			Multivariable^a^		
		Std *β*	SE	*P*-value	Std *β*	SE	*P*-value
Age	3802	− 0.22	0.02	** < 0.001**	− 0.12	0.02	** < 0.001**
Female sex	3802	− 0.08	0.03	** < 0.001**	− 0.08	0.02	** < 0.001**
BMI	3734	0.01	0.02	0.90			
Systolic BP	3774	0.09	0.02	** < 0.001**	0.04	0.02	**0.012**
Diastolic BP	3774	0.07	0.02	** < 0.001**			
Heart rate	3763	− 0.07	0.02	** < 0.001**			
HF characteristics							
Ischemic etiology	3257	0.12	0.04	** < 0.001**			
Years since HF diagnosis	1722	0.05	0.02	**0.026**			
Inpatient	3802	− 0.27	0.03	** < 0.001**			
Signs and symptoms							
NYHA class III/IV^b^	3737	− 0.15	0.03	** < 0.001**			
Peripheral edema	3252	− 0.15	0.03	** < 0.001**	− 0.08	0.03	** < 0.001**
Elevated JVP	2933	− 0.09	0.04	** < 0.001**			
Hepatomegaly	3640	− 0.11	0.05	** < 0.001**			
Pulmonary congestion	3668	− 0.17	0.03	** < 0.001**	− 0.04	0.03	**0.022**
Medical history							
Anemia	3704	− 0.13	0.03	** < 0.001**			
Atrial fibrillation	3788	− 0.04	0.03	**0.018**			
Hypertension	3787	0.01	0.03	0.63			
COPD	3787	− 0.02	0.04	0.23			
History of renal disease	3779	− 0.13	0.03	** < 0.001**	− 0.05	0.04	**0.013**
Stroke	3786	− 0.007	0.05	0.65			
PCI	3786	0.06	0.04	** < 0.001**	0.05	0.04	**0.003**
PAVD	3761	0.06	0.04	** < 0.001**	0.07	0.04	** < 0.001**
ACEi/ARB use at baseline	3802	0.09	0.04	** < 0.001**			
Laboratory parameters							
Hemoglobin	3704	0.08	0.02	** < 0.001**			
Leukocytes	3505	− 0.06	0.02	** < 0.001**			
C-reactive protein	2093	− 0.16	0.02	** < 0.001**			
Glucose	3144	− 0.01	0.02	0.53			
LDL-cholesterol	2014	0.02	0.02	0.42			
Sodium	3750	0.09	0.02	** < 0.001**			
Potassium	3741	0.02	0.02	0.25			
Albumin	3691	0.35	0.01	** < 0.001**	0.32	0.02	** < 0.001**
eGFR	3790	0.18	0.02	** < 0.001**	0.08	0.02	** < 0.001**
Creatinine	3792	− 0.20	0.02	** < 0.001**			
Urea	3523	− 0.25	0.02	** < 0.001**	− 0.11	0.02	** < 0.001**
NT-proBNP	3654	− 0.25	0.02	** < 0.001**	− 0.09	0.02	** < 0.001**
* NT-proBNP sinus*	1921	− 0.23	0.02	** < 0.001**			
* NT-proBNP AF*	1169	− 0.27	0.03	** < 0.001**			

Significant *P*-values are bold-printed

^a^*n* = 2783, Adjusted *R*^2^ = 0.276

^b^Compared to NYHA I/II as reference group

*ACEi* angiotensin-converting enzyme inhibitor, *ARB* angiotensin receptor blocker, *BMI* body mass index, *BP* blood pressure, *COPD* chronic obstructive pulmonary disease, *eGFR* estimated glomerular filtration rate, *JVP* jugular venous pressure, *LDL* low-density lipoprotein, *NT‐proBNP* N‐terminal pro‐B‐type natriuretic peptide, *NYHA* New York Heart Association, *PAVD* peripheral arterial vascular disease, *PCI* percutaneous coronary intervention

### Serum-free thiols and follow-up outcomes

**Fig. 1:**
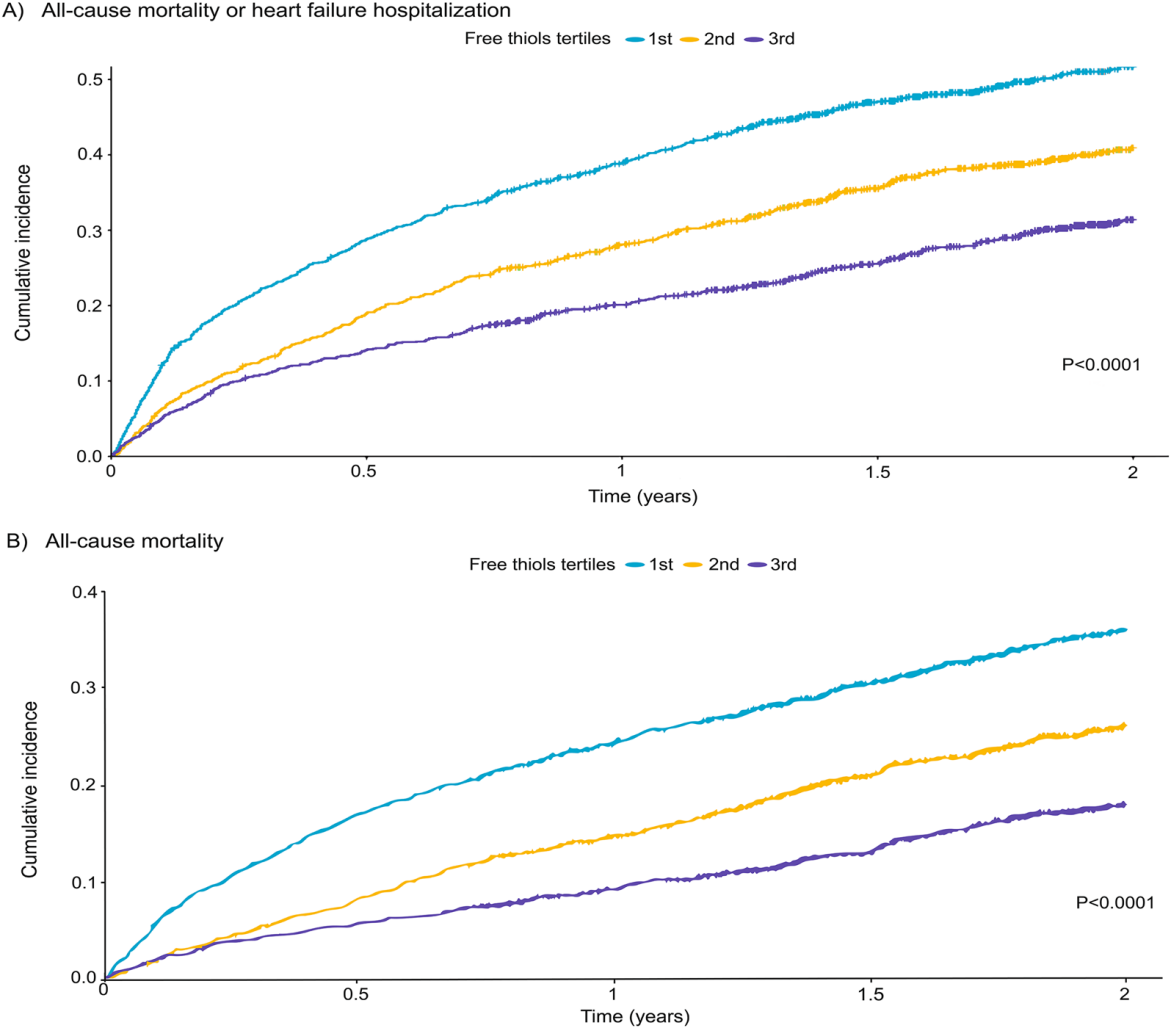
Cumulative incidence curves for the composite endpoint of all-cause mortality or heart failure-related hospitalizations at 2 years (**A**) and all-cause mortality at 2 years (**B**)

Median follow-up time of the combined study population was 545 [IQR 255–730] days. In the index cohort, median follow-up time was 553 [IQR 253–730] days, and for patients in the validation cohort, the median follow-up time was 517 [266–730] days. During 2 years of follow-up, 932 (24.5%) patients died, of which 594 (64%) due to a cardiovascular cause, and 908 (23.9%) patients were hospitalized for HF. The composite endpoint of all-cause mortality and HF hospitalization occurred in 1475 (38.8%) patients. Incidences of the composite outcome, as well as secondary outcome, differed significantly across tertiles (log-rank test: *P* < 0.001), and occurred most frequent in the lowest tertile of serum-free thiol levels (Fig. 1). In Cox regression analyses, lower levels of free thiols were associated with adverse disease outcome, also after adjustment for factors included in the BIOSTAT risk model [Hazard Ratio (HR) per SD decrease in free thiols: 1.058, 95%-confidence interval (CI): 1.001–1.118, *P* = 0.046 for the composite endpoint; Table 3]. Lower free thiol concentrations were also independently associated with higher rates of all-cause mortality [HR per SD decrease: 1.253, 95% CI: 1.171–1.341, *P* < 0.001] and cardiovascular mortality [HR per SD decrease: 1.182, 95% CI: 1.086–1.288, *P* < 0.001].

**Table 3:** Hazard ratios for serum-free thiol concentrations in predicting clinical endpoints

	Composite endpoint		All-cause mortality		Cardiovascular mortality^c^	
	HR per SD thiol decrease (95% CI)	*P*-value	HR per SD thiol decrease (95% CI)	*P*-value	HR per SD thiol decrease (95% CI)	*P*-value
Univariable	1.351 (1.283–1.422)	** < 0.001**	1.466 (1.374–1.565)	** < 0.001**	1.410 (1.300–1.530)	** < 0.001**
Age- and sex-adjusted	1.294 (1.227–1.365)	** < 0.001**	1.381 (1.291–1.477)	** < 0.001**	1.330 (1.225–1.450)	** < 0.001**
BIOSTAT-CHF risk model	1.058^a^ (1.001–1.118)	**0.046**	1.253^b^ (1.171–1.341)	** < 0.001**	1.182^b^ (1.086–1.288)	** < 0.001**

Significant *P*-values are bold-printed

*CI* confidence interval, *HF* heart failure, *HR* hazard ratio, *SD* standard deviation

^a^BIOSTAT-CHF risk model for composite endpoint (all-cause mortality & HF hospitalization): age, HF hospitalization in the year before inclusion, edema, N-terminal pro-B-type natriuretic peptide, systolic blood pressure, hemoglobin, high-density lipoprotein levels, serum sodium concentration and failure to prescribe a beta-blocker

^b^BIOSTAT-CHF risk model for predicting mortality: age, blood urea nitrogen, NT-proBNP, hemoglobin and the use of a beta-blocker at time of inclusion

^c^Non-cardiovascular mortality was used as competing risk

### Subgroup analyses

**Fig. 2:**
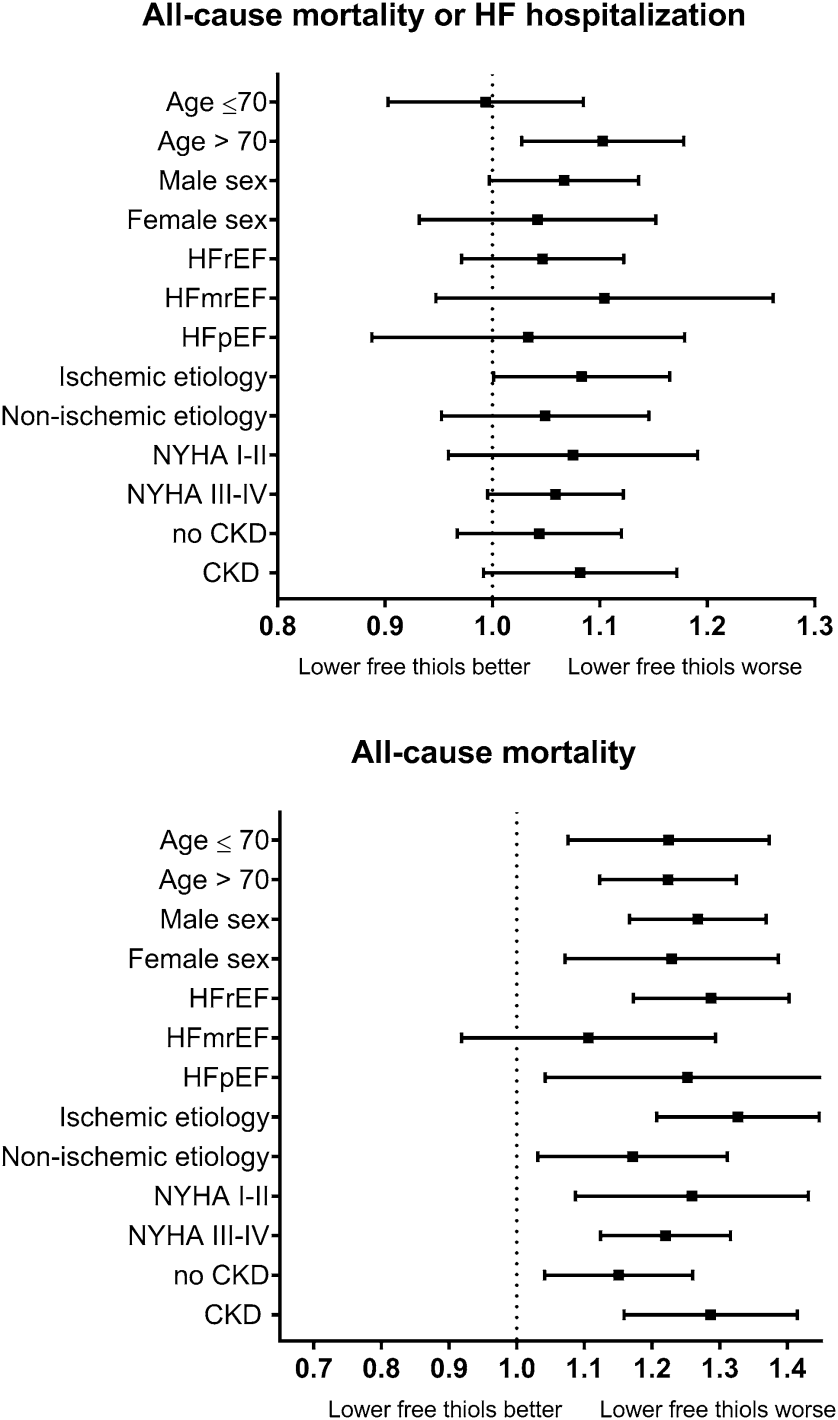
Forest plot depicting Hazard ratio’s for the composite endpoint of all-cause mortality or heart failure-related hospitalization (upper panel) and all-cause mortality alone (lower panel) during a 2-year follow-up, per SD decrease in serum-free thiols, across pre-specified subgroups. All Cox proportional hazards models were adjusted for the corresponding BIOSTAT-CHF risk model. Abbreviations: *CKD* chronic kidney disease, *HF* heart failure, *HFmrEF* heart failure with mildly reduced ejection fraction, *HFpEF* heart failure with preserved ejection fraction, *HFrEF* heart failure with reduced ejection fraction, *NYHA* New York Heart Association, *SD* standard deviation

Pre-defined subgroup analyses on the primary endpoint and on all-cause mortality were performed for age, sex, HF type, ischemic origin, NYHA class, and history of CKD. No significant subgroup interactions were observed (Fig. 2; Supplementary Table 3).

## Discussion

This study demonstrates that lower serum concentrations of free thiols, reflecting increased oxidative stress, are associated with greater HF severity and worse outcome for patients with new-onset or worsening HF. Our data provide rationale for future mechanistic studies on the role of free thiols in HF, and for studies evaluating effects of free thiol modulation for modifying disease outcomes for patients with HF.

Extracellular free thiols are critically involved in redox signaling, but also possess a strong antioxidant buffering capacity to a variety of reactive species [7]. Under conditions of oxidative stress, the circulating free thiol pool (reduced thiols with -SH group) scavenge ROS, leading to the oxidation of thiols and the formation of disulfide bonds. Therefore, measurement of the reduced, i.e. free, thiol pool is a simple, direct, and robust method to systemically assess the degree of oxidative stress [8].

Depletion of the free thiol pool has been observed and linked to disease severity in a variety of non-cardiovascular and cardiovascular diseases before, including anemia, diabetes mellitus, inflammatory bowel disease, kidney disease, and myocardial infarction [8, 9, 22–24], which was also investigated and confirmed for anemia and kidney disease in present study. In small exploratory studies including 120 and 101 patients with chronic HF, associations between serum-free thiol concentrations and increased HF severity [25, 26], and poorer clinical outcomes were suggested [26]. We substantiate previous work and extended upon this studies in several ways. Our sample size was almost 40 times larger reducing the risk of a type 1 error. Our study also generalized the link to HF by including patients with HFpEF, whereas the previous studies were limited to patients with a LVEF of < 45%. Furthermore, our patient population consisted of less-stable patients with new-onset or worsening HF on suboptimal pharmacological treatment, which were possibly more subjected to increased levels of oxidative stress. We observed robust and independent association between free thiol levels and disease outcomes, whereas the previous studies were underpowered to perform multivariable analyses on outcome. Our findings are consistent with the previous prognostic value of free thiols in a general population-based cohort [27, 28]. In the present study, we did not observe significant effect modifications between free thiols and disease outcomes by predefined subgroups.

Lower free thiol levels were consistently associated with signs of systemic or pulmonary congestion and natriuretic peptide levels. Other HF characteristics that were associated with lower free thiol levels were non-ischemic etiology, shorter duration of HF, inpatient study enrollment, and the absence of treatment with an ACEi or ARB. Differences in oxidative stress between ischemic vs dilated cardiomyopathy have been previously attributed to enhanced ROS-induced mitochondrial instability in the latter [29]. The activity of the antioxidant thioredoxin system was also higher in dilated cardiomyopathy, but it has been postulated this might be an indirect reflection of excessive oxidative stress [29, 30]. As for the time from HF diagnosis, we speculate that free thiols might be lower in the early phase of HF due to enhancement of ROS by catecholamines [31], and higher in a later phase due to the effect of pharmacological treatment. Participants that were enrolled as inpatients (acute HF) had likely more congestion and oxidative stress, resulting in lower free thiol levels. The association between the use of an ACEi or ARB might be associated with antioxidant effects of this class of therapy [32, 33], or might be coincidental due to the BIOSTAT-CHF design, which required participants to be on suboptimal pharmacological treatment. We cannot exclude that free thiol concentrations were also influenced by other factors, for example the production of thiol-containing molecules by the liver [7].

### Therapeutic potential

Associations between oxidative stress markers with heart failure severity and outcome have been established before, for example for 8-OHdG, malondialdehyde and uric acid [34, 35]. However, compared with other oxidative stress markers, free thiols constitute the central hubs of inter-organ redox communication [36], whereas malondialdehyde is merely a damage marker representing oxidative stress-induced lipid peroxidation. Moreover, free thiols are amendable for therapeutic modulation, for example by *N*-acetylcysteine or glutathione administration [10, 37, 38]. Our findings support the notion that thiol supplementation may have therapeutic potential in HF if a causal role of free thiols in the pathophysiology of HF can be addressed. Prior experimental studies showed that administration of N-acetylcysteine resulted in reduced oxidative stress and improved cardiac function in models with HF or ischemic injury [10–13, 39]. Beneficial effects of such thiol-containing drugs were also observed in patients with COVID-19 [40]. Thiol modulating studies in patients with HF are scarce and predominantly small or restricted to nitrate tolerance [14, 16, 41]. It has been suggested that administration of thiol-targeted antioxidants should be reserved for individuals with profound thiol depletion and incautious thiol supplementation could potentially disturb physiological redox signaling processes [42]. Therefore, more mechanistic studies and careful evaluation of treatment effects in patients with lower free thiols, are warranted. In this respect, circulating free thiols may aid in patient stratification to restore the systemic and local redox balance following a redox precision medicine approach.

### Strengths and limitations

Strengths of our study include the large sample size, the phenotypically well-characterized patient population, combined with a systematic follow-up. Limitations of our study include the geographic restrictions of our cohort recruiting mainly Caucasians since some studies suggest ethnic and geographic differences in oxidative stress-related gene expression and antioxidant status [43, 44]. We also did not have data on dietary intake or total serum protein levels to make further adjustment for serum-free thiol level [7, 36]. Moreover, participants were enrolled back in 2010–2012, and HF treatments have advanced since then. At last, our results do not prove causality or mechanisms of free thiol depletion.

## Conclusions

In patients with new-onset or worsening HF lower serum-free thiol concentrations, indicative of increased oxidative stress, are associated with greater HF severity and a poorer prognosis. If future studies prove a causal role of free thiols in the progression of HF, it might be of interest to study whether free thiol modulation, especially in patients with poor redox status, might improve clinical outcomes.

## Supplementary Information

Below is the link to the electronic supplementary material.

Supplementary file1 (PDF 209 KB)

## Data Availability

The data underlying this article will be shared on reasonable request to the corresponding author.
