# Supplementary material for: Systemic oxidative stress associates with disease severity and outcome in patients with new-onset or worsening heart failure
Source: Clin Res Cardiol. 2023 Mar 30;112(8):1056–66. doi: 10.1007/s00392-023-02171-x (PMC10062262; doi:10.1007/s00392-023-02171-x)
Supplement: Supplementary file 1 — Supplementary file1 (PDF 209 KB) [file 392_2023_2171_MOESM1_ESM.pdf]

Supplementary information

Title: Systemic oxidative stress associates with disease severity and outcome in patients with new-onset or worsening heart failure

Authors: Marie-Sophie L.Y. de Koning, Johanna E. Emmens, Esteban Romero-Hernández, Arno R. Bourgonje, Solmaz Assa, Sylwia M. Figarska, John GF Cleland, Nilesh J. Samani, Leong L. Ng, Chim C. Lang, Marco Metra, Gerasimos S. Filippatos, Dirk J. van Veldhuisen, Stefan D. Anker, Kenneth Dickstein, Adriaan A. Voors, Erik Lipsic, Harry van Goor, and Pim van der Harst

Corresponding Author Contact: M.L.Y. de Koning. University of Groningen, University Medical Center Groningen, Department of Cardiology, Groningen, the Netherlands, Hanzeplein 1, PO Box 30.001, 9700 RB, Groningen, the Netherlands. E-mail: m.s.l.y.de.koning@umcg.nl

Journal: Clinical Research in Cardiology

Content:

	Page
Supplementary Table 1	2
Supplementary Table 2	4
Supplementary Table 3	6

Supplementary Table 1 – Baseline characteristics of the BIOSTAT-CHF index cohort according to tertiles of serum free thiols

	1st tertile n=726	2nd tertile n=725	3rd tertile n=725	*P*-value
Serum free thiols (µmol/L)	227 [190;258] Full range (38-281)	326 [304;348] Full range (281-369)	419 [392;458] Full range (369-799)	
Demographics				
Age (years)	74.2 [65.7;79.8]	70.7 [62.1;78.8]	65.2 [56.6;74.1]	**<0.001**
Female sex	237 (33%)	208 (29%)	142 (20%)	**<0.001**
BMI (kg/m^2)	26.7 [23.6;30.5]	27.2 [23.9;30.5]	27.2 [24.5;30.9]	0.198
HF type				**<0.001**
HFrEF	503 (78%)	521 (80%)	549 (84%)	
HFmrEF	82 (13%)	81 (13%)	78 (12%)	
HFpEF	64 (10%)	46 (7%)	24 (4%)	
Months since HF diagnosis	1 [0;12]	6 [0;46]	3 [0;53]	0.305
Ischaemic etiology	304 (43%)	330 (46%)	319 (45%)	0.421
Inpatient enrolment	604 (83%)	520 (72%)	423 (58%)	**<0.001**
NYHA class				**<0.001**
I/II	207 (30%)	269 (38%)	332 (47%)	
III/VI	494 (71%)	431 (62%)	379 (53%)	
Systolic BP (mmHg)	120 [110;135]	120 [110;140]	120 [110;136]	**0.019**
Diastolic BP (mmHg)	70 [64;80]	73 [66;82]	76.0 [70;85]	**<0.001**
Heart rate (bpm)	77 [66;90]	76 [68;90]	75 [67;88]	0.854
LVEF (%)	30 [25;38]	30 [25;36]	30 [25;35]	**0.038**
Signs and symptoms				
Peripheral oedema	433 (67%)	352 (60%)	288 (51%)	**<0.001**
Elevated JVP	208 (39%)	169 (32%)	116 (23%)	**<0.001**
Hepatomegaly	127 (18%)	101 (14%)	81 (11%)	**0.002**
Pulmonary congestion	444 (63%)	393 (56%)	301 (43%)	**<0.001**
Medical history				
Anaemia	304 (43%)	240 (35%)	220 (32%)	**<0.001**
Atrial fibrillation	369 (51%)	340 (47%)	282 (39%)	**<0.001**
Diabetes Mellitus	233 (32%)	235 (32%)	234 (32%)	0.991
COPD	139 (19%)	124 (17%)	112 (15%)	0.175
CKD	282 (39%)	186 (26%)	144 (20%)	**<0.001**
Hypertension	460 (63%)	459 (63%)	435 (60%)	0.318
PAVD	84 (12%)	81 (11%)	81 (11%)	0.963
Stroke	78 (11%)	73 (10%)	54 (7%)	0.076
PCI	128 (18%)	140 (19%)	179 (25%)	**0.002**
CABG	134 (19%)	133 (18%)	105 (15%)	0.073
Medication use at baseline				
Loop diuretics	723 (99%)	722 (99%)	719 (99%)	0.585
Loop diuretic dose (mg furosemide equivalent)	40 [40;125]	40 [40;100]	40 [40;80]	**<0.001**
ACEi/ARB	491 (68%)	517 (71%)	556 (77%)	**0.001**
Beta-blocker	595 (82%)	604 (83%)	608 (84%)	0.609
Aldosteron antagonist	376 (52%)	382 (53%)	383 (53%)	0.912
Laboratory				
Haemoglobin (g/dL)	12.9 [11.6;14.1]	13.4 [12.0;14.5]	13.6 [12.2;14.8]	**<0.001**
Leukocytes (10^9/L)	7.8[6.3;9.8]	7.9 [6.6;9.6]	7.8 [6.4;9.4]	0.581
Sodium (mmol/L)	139 [136;142]	140 [137;142]	140 [137;142]	0.143
Potassium (mmol/L)	4.2 [3.8;4.6]	4.2 [3.9;4.6]	4.3 [3.9;4.6]	0.092
Urea (mmol/L)	13.6 [8.6; 22.5]	10.5 [7.3; 16.1]	9.8 [7.0; 15.3]	**<0.001**
Serum creatinin (µmol/L)	113 [90;150]	103 [82;128]	97 [80;116]	**<0.001**
eGFR (mL/min/1.73 m^2)	51 [35;68]	59 [45;77]	68 [52;84]	**<0.001**
NT-proBNP (ng/L)	3890 [1836; 8325]	2858 [1329;5386]	1910 [851;4052]	**<0.001**
Albumin (g/L)	29 [24;34]	32 [27;37]	36 [31;41]	**<0.001**

LDL-cholesterol (mmol/L)	2.2 [1.7; 3.0]	2.6 [1.9; 3.3]	2.6 [2.0; 3.3]	**<0.001**
HDL-cholesterol (mmol/L)	1.02 [0.83;1.30]	1.06 [0.88;1.32]	1.04 [0.85;1.30]	0.753
Glucose (mmol/L)	6.3 [5.4;7.8]	6.4 [5.4;8.0]	6.2 [5.4;8.0]	0.539

Data shown as median [IQR] or n (%). Significant *P*-values are bold-printed.
ACEi, angiotensin-converting enzyme inhibitor; ARB, angiotensin receptor blocker; BMI, body mass index; BP, blood pressure; CABG, coronary artery bypass graft; COPD, chronic obstructive pulmonary disease; CKD, chronic kidney disease; eGFR, estimated glomerular filtration rate; HbA1c, glycated haemoglobin; HDL, high-density lipoprotein; HF, heart failure; HFmrEF, heart failure with mid-range ejection fraction; HFpEF, heart failure with preserved ejection fraction; HFrEF, heart failure with reduced ejection fraction; JVP, jugular venous pressure; LDL, low-density lipoprotein; LVEF, left ventricular ejection fraction; NT-proBNP, N-terminal pro-B-type natriuretic peptide; NYHA, New York Heart Association; PAVD, peripheral arterial vascular disease; PCI, percutaneous coronary intervention.

Supplementary Table 2 – Baseline characteristics of the BIOSTAT-CHF validation cohort according to tertiles of serum free thiols

	1st tertile n=542	2nd tertile n=542	3rd tertile n=542	*P*-value
Serum free thiols (μmol/L)	269 [236;296] Full range (59-317)	353 [337;371] Full range (317-388)	431 [406;461] Full range (388-645)	
Demographics				
Age (years)	78.2 [70.6;83.9]	75.9 [68.3;81.6]	70.8 [64.5;78.0]	**<0.001**
Female sex	215 (40%)	198 (37%)	146 (27%)	**<0.001**
BMI (kg/m^2)	27.5 [23.9;32.1]	28.0 [24.8;32.7]	28.5 [25.1;32.7]	0.051
HF type				0.580
HFrEF	219 (44%)	234 (48%)	220 (46%)	
HFmrEF	130 (26%)	123 (25%)	133 (28%)	
HFpEF	150 (30%)	131 (27%)	127 (27%)	
Months since HF diagnosis	11 [0.2;48]	20 [2;63]	16 [2;58]	**<0.001**
Inpatient enrolment	377 (70%)	281 (52%)	216 (40%)	**<0.001**
Ischaemic etiology	354 (94%)	360 (94%)	339 (94%)	0.935
NYHA class				**<0.001**
I/II	163(30%)	221 (41%)	298 (55%)	
III/VI	379 (70%)	321 (59%)	243 (45%)	
Systolic BP (mmHg)	118 [106;136]	126 [113;141]	125 [112;144]	**<0.001**
Diastolic BP (mmHg)	65 [57;74]	69 [60;78]	70 [63;79]	**<0.001**
Heart rate (bpm)	73 [63;85]	70 [60;84]	72 [63;83]	0.210
LVEF (%)	41 [35;50]	40 [33;50]	40 [35;50]	0.269
Signs and symptoms				
Peripheral oedema	368 (76%)	300 (61%)	229 (49%)	**<0.001**
Elevated JVP	170 (37%)	131 (28%)	113 (25%)	**<0.001**
Hepatomegaly	24 (5%)	14 (3%)	16 (3%)	0.202
Pulmonary congestion	297 (58%)	230 (44%)	160 (31%)	**<0.001**
Medical history				
Anemia	274 (51%)	199 (37%)	130 (24%)	**<0.001**
Atrial fibrillation	244 (45%)	238 (44%)	229 (43%)	0.716
Diabetes Mellitus	190 (35%)	175 (32%)	166 (31%)	0.278
COPD	113 (21%)	95 (18%)	85 (16%)	0.079
CKD	319 (59%)	258 (48%)	161 (31%)	**<0.001**
Hypertension	319 (59%)	317 (59%)	306 (57%)	0.673
PAVD	103 (20%)	111 (21%)	137 (26%)	**0.033**
Stroke	114 (21%)	98 (18%)	83 (16%)	0.052
PCI	92 (17%)	102 (19%)	113 (21%)	0.265
CABG	78 (14%)	99 (18%)	106 (20%)	0.067
Medication use at baseline				
Loop diuretics	535 (99%)	535 (99%)	537 (99%)	0.808
Loop diuretic dose (mg furosemide equivalent)	80 [40;100]	40.0 [40;80]	40 [40;80]	**<0.001**
ACEi/ARB	359 (66%)	374 (69%)	421 (78%)	**<0.001**
Beta-blocker	372 (69%)	394 (73%)	418 (77%)	**0.007**
Aldosteron antagonist	170 (31%)	179 (33%)	175 (32%)	0.842
Laboratory				
Haemoglobin (g/dL)	12.5 [11.1;13.8]	13.2 [11.8;14.5]	13.9 [12.6;15.1]	**<0.001**
Leukocytes (10^9/L)	7.7 [6.1;9.6]	7.4 [6.1;9.1]	7.3 [6.1;8.9]	**0.019**
Sodium (mmol/L)	139 [136;141]	140 [137;141]	140 [138;141]	**<0.001**
Potassium (mmol/L)	4.3 [4.0;4.6]	4.3 [4.0;4.6]	4.3 [4.0;4.6]	0.857
Urea (mmol/L)	10.0 [7.5;14.9]	8.6 [6.8;11.6]	7.5 [5.9;9.8]	**<0.001**
Serum creatinin (μmol/L)	108 [84;142]	96 [80;124]	89 [76;111]	**<0.001**
eGFR (mL/min/1.73 m^2)	54 [38;60]	60 [44;60]	60 [55;60]	**<0.001**
NT-proBNP (pg/mL)	2448 [958;6447]	1473 [554;3210]	828 [294;1863]	**<0.001**
Albumin (g/L)	35 [31;39]	39 [35;43]	39 [36;43]	**<0.001**

LDL-cholesterol (mmol/L)	1.9 [1.5;2.3]	2.0 [1.5;2.5]	2.0 [1.5;2.6]	0.080
HDL-cholesterol (mmol/L)	1.09 [0.86;1.37]	1.09 [0.90;1.35]	1.10 [0.89;1.40]	0.652
Glucose (mmol/L)	6.7 [5.5;8.7]	6.2 [5.2;8.3]	5.9 [5.1;8.2]	**0.001**

Data shown as median [IQR] or n (%). Significant *P*-values are bold-printed.
ACEi, angiotensin-converting enzyme inhibitor; ARB, angiotensin receptor blocker; BMI, body mass index; BP, blood pressure; CABG, coronary artery bypass graft; COPD, chronic obstructive pulmonary disease; CKD, chronic kidney disease; eGFR, estimated glomerular filtration rate; HbA1c, glycated haemoglobin; HDL, high-density lipoprotein; HF, heart failure; HFmrEF, heart failure with mid-range ejection fraction; HFpEF, heart failure with preserved ejection fraction; HFrEF, heart failure with reduced ejection fraction; JVP, jugular venous pressure; LDL, low-density lipoprotein; LVEF, left ventricular ejection fraction; NT-proBNP, N-terminal pro-B-type natriuretic peptide; NYHA, New York Heart Association; PAVD, peripheral arterial vascular disease; PCI, percutaneous coronary intervention.

Supplementary Table 1 - Risk model-adjusted hazard ratios of serum free thiols in predicting the composite endpoint (all-cause mortality or heart failure hospitalization) and all-cause mortality in 2 years across pre-specified subgroups

Variable		*Composite endpoint* [c]		*All-cause mortality* [d]	
	n	**HR per SD decrease (95% CI)**	***P* for interaction**	**HR per SD decrease (95% CI)**	***P* for interaction**
**Age**			0.34		0.25
≤ 70	1592	0.991 (0.904 – 1.086)		1.218 (1.079 – 1.376)	
> 70	2203	1.101 (1.028 – 1.179)		1.221 (1.124 – 1.326)	
**Sex**			0.62		0.79
Male	2650	1.065 (0.998 – 1.137)		1.265 (1.168 – 1.370)	
Female	1145	1.038 (0.934 – 1.154)		1.222 (1.075 – 1.390)	
**HF groups**			0.76 [a]<br>0.48 [b]		0.16 [a]<br>0.49 [b]
HFrEF	2246	1.045 (0.972 – 1.123)		1.284 (1.174 – 1.404)	
HFmrEF	627	1.097 (0.951 – 1.265)		1.095 (0.924 – 1.299)	
HFpEF	542	1.027 (0.891 – 1.182)		1.241 (1.048 – 1.468)	
**Ischemic etiology**			0.82		0.71
Yes	2000	1.081 (1.002 – 1.166)		1.323 (1.209 – 1.449)	
No	1251	1.046 (0.954 – 1.147)		1.166 (1.034 – 1.314)	
**NYHA class**			0.18		0.29
I-II	1488	1.071 (0.961 – 1.193)		1.251 (1.091 – 1.435)	
III-IV	2242	1.054 (0.988 – 1.124)		1.218 (1.125 – 1.317)	
**History of renal disease**			0.71		0.64
Yes	1347	1.079 (0.993 – 1.173)		1.282 (1.161 – 1.417)	
No	2425	1.042 (0.968 – 1.121)		1.147 (1.043 – 1.262)	

[a] HFmrEF compared to HFrEF
[b] HFpEF compared to HFrEF
[c] BIOSTAT-CHF risk model for composite endpoint (all-cause mortality & HF hospitalization): age, heart failure hospitalization in the year before inclusion, oedema, N-terminal pro-B-type natriuretic peptide, systolic blood pressure, haemoglobin, high-density lipoprotein levels, serum sodium concentration and failure to prescribe a beta-blocker.
[d] BIOSTAT-CHF risk model for predicting all-cause mortality: age, blood urea nitrogen, NT-proBNP, haemoglobin and the use of a beta-blocker at time of inclusion.
CI, confidence interval; HF, heart failure; HFmrEF, heart failure with mildly reduced ejection fraction; HFpEF, heart failure with preserved ejection fraction; HFrEF, heart failure with reduced ejection fraction; HR, hazard ratio; NYHA, New York Heart Association; SD standard deviation.
